
JOURNAL INFORMATION
==============================
NLM Title Abbreviation: ScientificWorldJournal
Journal ID: ScientificWorldJournal
NLM Journal ID: 101131163
Journal ID: TSWJ

The Scientific World Journal

ISSN: 2356-6140
EISSN: 1537-744X
Publisher: Wiley

ARTICLE INFORMATION
==============================
PMCID: PMC6009227
PMID: 29973858
DOI: 10.1100/tsw.2002.881
Article ID: 260157
Article version: 1
Subjects: Editorial

The Marine Systems Domain of TheScientificWorldJOURNAL

Richert Simon
Publication date: 2002
Electronic publication date: 2002 Jun 27
Volume: 2
First page: 1774
Last page: 1775
Received 2002 Jun 17; Accepted 2002 Jun 17
Copyright: Copyright © 2002 Simon�Richert.
Copyright year: 2002
License: This is an open access article distributed under the Creative Commons Attribution License, which permits unrestricted use, distribution, and reproduction in any medium, provided the original work is properly cited.
License URL: https://creativecommons.org/licenses/by/3.0/

==============================
**Publisher's Note**
*TheScientificWorldJOURNAL* (2002) 2, 1774–1775
ISSN 1537-744X; DOI 10.1100/tsw.2002.881

### The Marine Systems Domain of *TheScientificWorldJOURNAL*

Simon Richert
*Editor, TheScientificWorldJOURNAL*

E-mail: srichert@thescientificworld.com

*Received June 17, 2002; Accepted June 17, 2002; Published June 27, 2002*

**DOMAIN:** marine systems

As the Publishing Editor responsible for all Domains related to the marine sciences in *TheScientificWorldJOURNAL*, I am delighted to be developing the Marine Systems Domain. This Domain has a broad environmental focus, and encourages a range of submissions that are all subject to thorough peer review.

The quality of content is ensured by the appointment of a distinguished international editorial panel that represents a range of research interests. The Principal Editor of the Domain is Dr. Charles Sheppard at the University of Warwick (U.K.), who will be assisted by Associate Editor Dr. Richard Kenchington (RAC Marine PTY Ltd, Australia). Appointments to the Editorial Board are based on each candidate's research interests, and the creation of a diverse board with a strong geographical and gender representation has been pursued. Board members are currently based in the U.S., Canada, Italy, Germany, Hong Kong, Australia, Monaco, and the U.K., and further Editors will be appointed in due course.

The Marine Systems Domain hopes to encourage a wide range of papers that will be dealt with efficiently and published promptly. The Journal's division into Domains, each of which is the equivalent of a peer-reviewed journal and has its own Editorial Board, whilst being fully networked to form a "Journal of many Journals", allows the widest possible exposure of papers. This unique structure allows the multidisciplinary exchange of information by virtue of papers being published in all related Domains. Papers submitted to the Marine Systems Domain therefore can be published in a range of other Domains, and we envisage that the Domain will work closely with other related Domains including Oceans Biology, Freshwater Systems, and Domains devoted to the areas of Environmental Science and Management.

*TheScientificWorldJOURNAL* and the Editors of the Marine Systems Domain look forward to developing its coverage, and hope that it becomes the premier venue for publishing cutting edge science in the field of marine environmental systems. The digital nature of the Journal and the flexible options that it offers authors enables it to capture the benefits of the internet — unhampered by restrictions of print and offering immediate global dissemination. The future of marine science publishing has arrived!

©2002 with author.

**This article should be referenced as follows:**

Richert, S. (2002) The Marine Systems domain of *TheScientificWorldJOURNAL*. *TheScientificWorldJOURNAL* **2**, 1774–1775.
